# Cryo‐EM Analysis in CASP16

**DOI:** 10.1002/prot.70099

**Published:** 2025-12-11

**Authors:** Thomas Mulvaney, Andriy Kryshtafovych, Maya Topf

**Affiliations:** ^1^ Research Department of Integrative Virology Leibniz Institute of Virology (LIV) Hamburg Germany; ^2^ Center for Structural Systems Biology (CSSB), DESY Hamburg Germany; ^3^ Genome Center University of California Davis California USA; ^4^ Institute for Molecular Virology and Tumorvirology University Medical Center Hamburg‐Eppendorf (UKE) Hamburg Germany

**Keywords:** CASP, cryo‐EM, flexibility, local resolution, protein structure, RNA structure, structure prediction

## Abstract

Since CASP13, experimentalists have been encouraged to provide their cryo‐EM data along with the derived atomic models to the CASP organizers to aid assessment. In CASP16, 38 cryo‐EM datasets were provided for assessment, which represented most cryo‐EM targets. The corresponding targets typically comprised a single derived atomic structure; however, that model may be only one of several valid conformations. Flexibility often manifests as low‐resolution regions in a cryo‐EM reconstruction, particularly in RNA but often also in protein complexes. We show that local resolution in the reconstruction correlates well with the root‐mean‐square fluctuations (RMSF) of residues of accurate CASP predictions. The correlation between the local resolution and pLDDT was less clear, especially when mobile domains were present. When the resolution allowed, assessment of features such as sidechains, using our variant of SMOC with local fragment alignment, indicated that even high‐quality predictions have room for improvement; on the other hand, some predictions fitted the density better in specific regions, indicating modeling discrepancies in the target. In one extreme case, a submitted target had regions of low‐resolution that limited unambiguous model building. In such cases, part of the target structure is essentially a prediction itself, with implications for the assessment. Experimental data remain essential for model‐free assessment of predictions and offer unique analyses such as comparisons to local resolution and thus flexibility.

## 1 Introduction

Cryo‐electron microscopy has become an important technique for structure determination, providing structural biologists with insights into molecules that were previously unobtainable through crystallographic methods. The Critical Assessment of Structure Prediction (CASP) community has benefited heavily from a stream of cryo‐EM targets that are typically larger than crystallographic targets and oftentimes contain folds that are under‐represented in structural databases such as the PDB [1].

**FIGURE 1:**
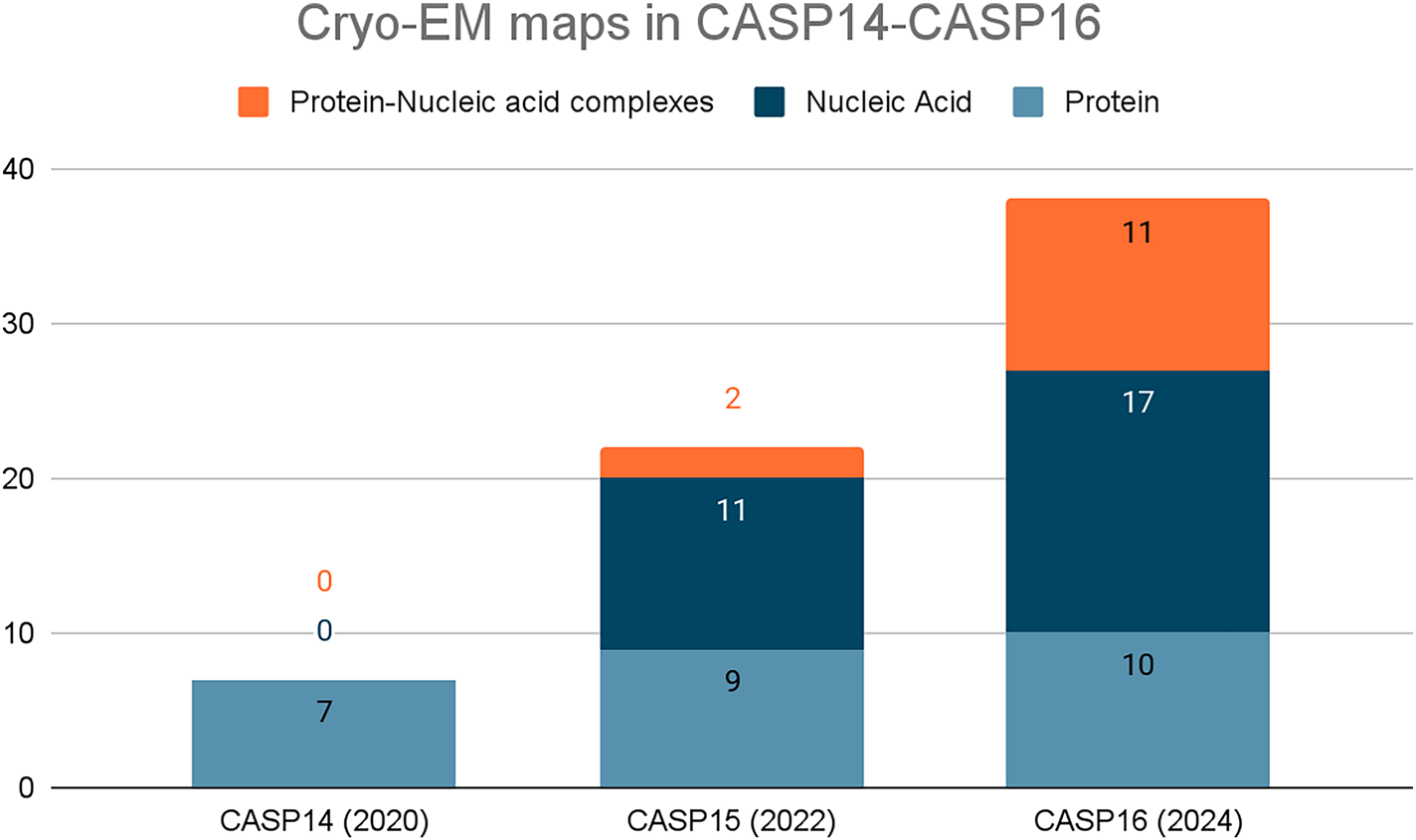
The number of cryo‐EM datasets being provided for assessment has increased steadily, with cryo‐EM proving to be an important technique for solving RNA and hybrid structures.

Since 2020, the number of cryo‐EM targets in CASP has been steadily increasing (Figure 1), mirroring the PDB deposition tendency. In this CASP, 49 of the targets (corresponding to 47 maps; T1234 and T1235 were derived from the same map as the complex, H1236) were obtained from cryo‐EM experiments, with X‐ray crystallography and NMR providing 48 and two targets respectively. Thus, for the first time in CASP, the number of targets from cryo‐EM has caught up with that of X‐ray crystallography. Predictions for cryo‐EM targets are assessed against the target model, similar to how crystallographic and NMR targets are assessed, using standard metrics of accuracy. Our previous papers in this series [2, 3, 4] explored the possibility of validation of CASP models versus the cryo‐EM data using both local and global goodness‐of‐fit measures. In CASP15, we showed that the fit‐to‐map‐based ranking correlates well with the CASP assessment scores [4]. Additionally, by performing a local assessment of predicted sidechains, we found that they are sometimes poorly positioned in models.

Structure prediction continues to play an important role in interpreting low‐resolution cryo‐EM data, especially in cases where the resolution is insufficient to build a complete atomic model de novo. One popular approach to model building with a structural prior is *flexible fitting* and *refinement* [5, 6], where an initial model (potentially a prediction) is adjusted to better fit the density. In CASP14, we demonstrated that state‐of‐the‐art predictions (notably those from AlphaFold2 but also others) can automatically be refined into very accurate models, reaching the accuracy of the structure provided by the experimentalists [3]. We also investigated variations between different predictions of single chains by different groups and correlated these with the quality of local fit in the map. With the improvement in complex prediction and the introduction of RNA targets, refinement in CASP15 shifted to entire protein complexes [4] rather than individual domains. By combining TEMPy‐ReFF [7], RIBFIND [8], and, in the case of RNA models, ERRASER2 [9], this refinement pipeline was often able to produce models of equal or better fit to the experimental maps than the targets provided by experimentalists.

**FIGURE 2:**
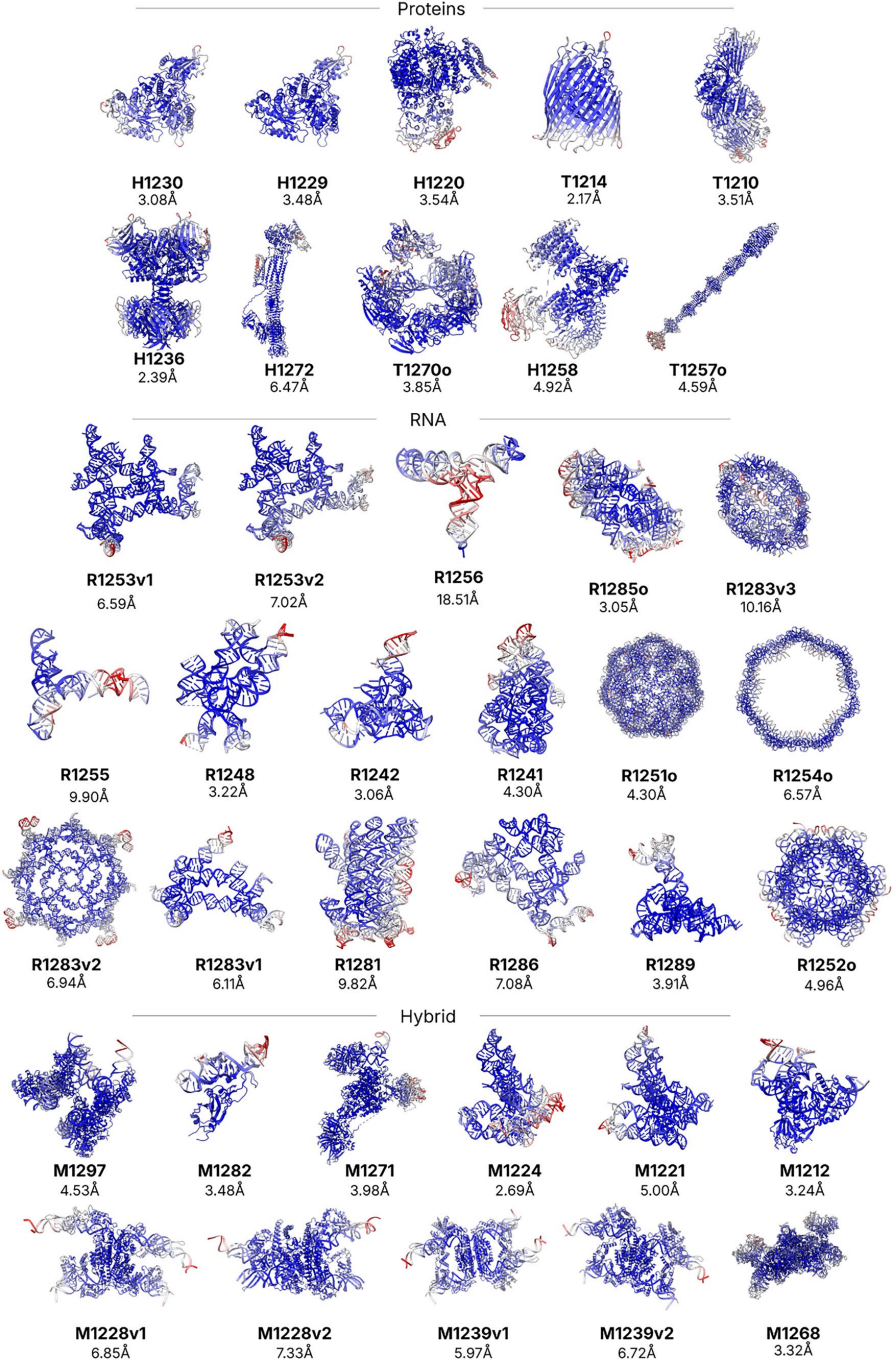
Overview of cryo‐EM targets colored by local resolution. Targets are colored according to local resolution (LocRes). Low‐resolution is red and high‐resolution is blue. The distributions of these resolutions are provided in Figure 3A and mean and standard deviation for each target are provided in Table S1.

In CASP16, of the 47 cryo‐EM determined structures, 38 maps were provided for assessment (Figure 2). For the first time, we requested that experimentalists provide the unprocessed half‐maps, in line with established best practices in the cryo‐EM community [10]. The requested half‐maps allowed additional types of assessment, which were previously not feasible, such as local resolution estimation. This, in turn, the allowed for new avenues of inquiry, such as whether predictions are able to capture this information. By analyzing multiple submissions, we aimed to capture hints of conformational dynamics or alternative local arrangements indicated by the cryo‐EM data, essentially asking whether predictors' model diversity reflects regions of uncertainty or flexibility. This allows us to see if regions of a target that were poorly resolved in the cryo‐EM map (and thus presumably more flexible or uncertain) also showed larger disagreement across predictions, suggesting that the prediction ensembles can encode experimental dynamics information beyond what a single target model provides.

Finally, the interplay between prediction and experiment in CASP has directly helped structure determination. In CASP14, several targets that could not be solved in time were ultimately resolved with the aid of high‐quality predictions from AlphaFold2 [11, 12]. Likewise, in CASP16, there were three cases where clear modeling errors in the initially provided “solved” structures were detected and corrected with the aid of accurate high‐quality structure predictions (T1210, H1220, T1257o). These errors would otherwise have gone unnoticed had the authors not provided their experimental cryo‐EM data and engaged with the prediction community. In this paper, we investigate these and other cases in greater detail, employing a local goodness‐of‐fit metric to the cryo‐EM density to systematically identify and analyze regions where predictions and experimental data diverge.

## 2 Methods

### 2.1 Experimental Data Collection and Participation

As mentioned above, experimentalists provided the unprocessed half‐maps of all 38 cryoEM maps. Datasets covered a range of target types, including 10 protein complexes, 17 RNA structures, both synthetic and natural [13], and 11 hybrid targets composed of proteins and nucleic acids. Due to the size of the targets or the presence of multiple domains or units of interest, some were further broken down to yield additional targets for assessment, such as H1236, which was split into T1234 and T1235 as evaluation units. Across these different targets, the resolutions ranged significantly (Table S1, Figure 2). Generally, protein structures and complexes had higher resolution than RNA and hybrid targets. Nucleic acid‐containing structures have historically suffered from lower‐resolutions, and only in recent years have the advances in cryo‐EM technology and methodology enabled resolutions sufficient for deriving atomic models. The lowest resolution CASP16 targets were single‐chain RNA structures from SARS‐2 (R1255 and R1256). Compared with the previous CASP, which had only two RNA‐protein targets [14], this CASP saw a huge increase in hybrid targets, many solved by cryo‐EM. Unlike in previous CASP rounds, in this round we requested experimentalists to provide the half‐maps for their cryo‐EM reconstructions. This was in part a response to reconstructions received in CASP15, which had been post‐processed using deep learning‐based sharpening tools such as DeepEMhancer. Such sharpening approaches are often useful aids in model building, but they have a tendency to make some features, such as ligands, disappear. Local resolution fluctuations are also flattened. Such information can be distracting for model building but are often indications of heterogeneity and mobility, features which might make structure prediction difficult and interpretation challenging.

### 2.2 Local Resolution Determination

The Relion 3 software [15] was used to prepare local resolution maps from the provided half‐maps, referred to here as “LocRes.” In local resolution maps, the value of each voxel corresponds to the estimated resolution at that coordinate. The local resolution was then projected onto the experimental model by taking the determined resolution of the voxel closest to each atom. These local resolution projections are presented in Figure 2. The average local resolution for each structure is the average of all‐atom local resolutions and is shown beneath the targets in Figure 2 and Table S1. (Note that this is not calculated on the entire map but only on regions occupied by atoms).

**FIGURE 3:**
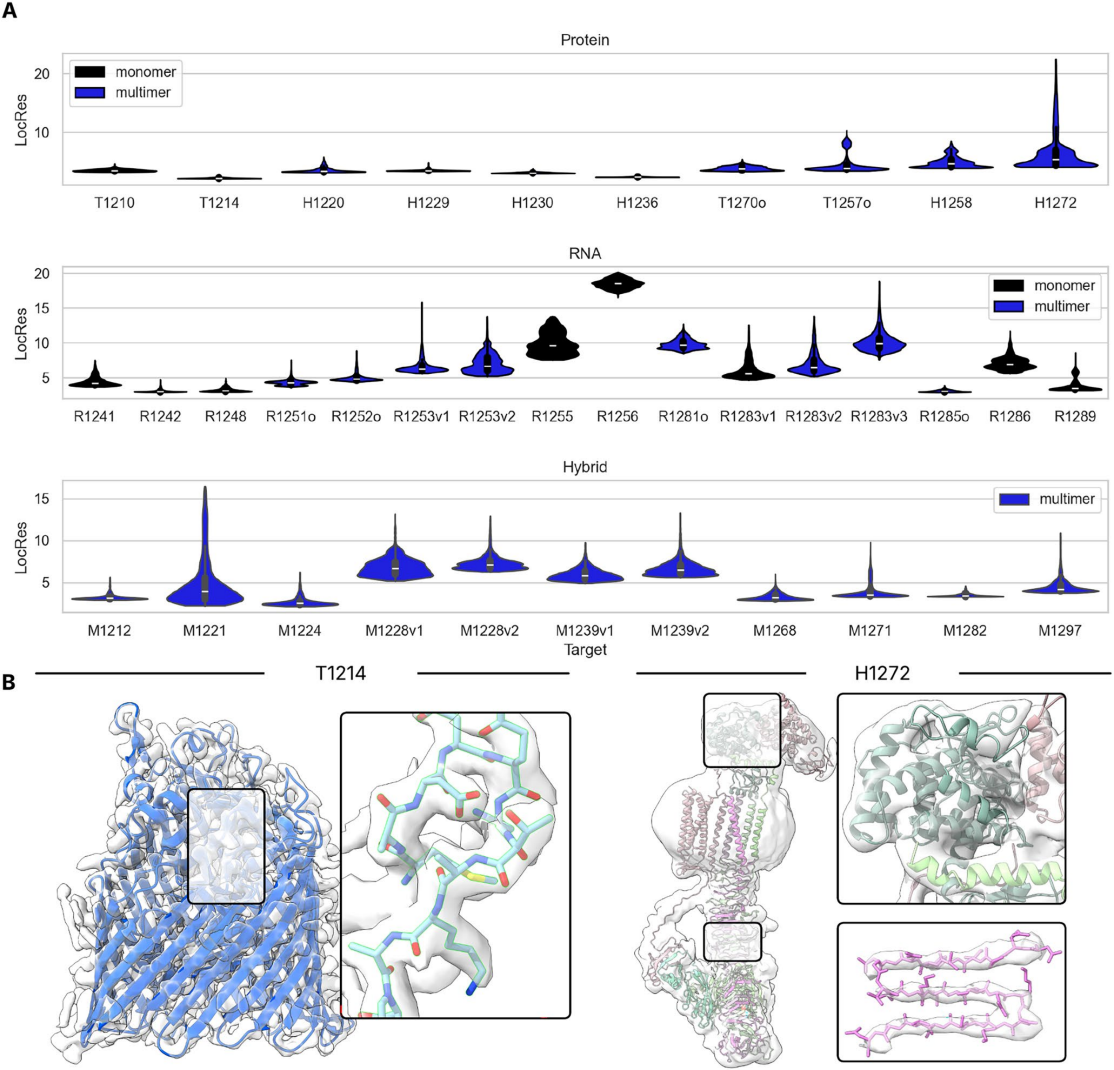
Overview of cryo‐EM local resolutions for targets. (A) The distribution of local resolutions is given for each target. In (B) a close‐up of visible details for two targets is shown. On the left, T1214, which had an average resolution of 2.17 Å, with sidechains well resolved throughout the reconstruction. On the right H1272, which had an average resolution of 6.47 Å but varied significantly throughout the structure. In one region, the backbone of beta sheets is nicely resolved, but in other regions, even larger secondary structures were not well resolved.

The mean local resolution of the reconstructions for 10 protein targets varied from 2.17 Å to 6.47 Å. Examples of the structural details present at these two extremes are shown in Figure 3B. T1214 has visible density for sidechains throughout the molecule. On the other hand, H1272 has a high‐resolution core with visible backbone density around beta sheets, but other regions lack backbone visibility. Due to this low‐resolution, some parts of this target structure were built by rigidly docking AlphaFold 2 and 3 models into the density. Heterogeneity in the resolution of cryo‐EM reconstructions can be explained in part by the conformational flexibility of the molecules being imaged. As is often expected, nucleic acid‐containing structures had lower mean resolutions, ranging from 3.05 Å and extending down to 18.51 Å. The average of these mean resolutions for RNA targets was 6.99 Å. Hybrid targets had mean local resolutions between 2.69 Å and 7.32 Å. The average mean resolution was 4.83 Å.

Careful alignment and classification of the molecular images (“particles”) can help ensure that the reconstructions come from images of molecules with similar conformations. In some datasets, conformationally distinct classes were captured, as in the case of M1228 and M1239. These classes were reconstructed individually, offering targets in alternative states: M1228 v1 and v2, and M1239 v1 and v2. Still, smaller variations are difficult to separate into individual reconstructions, yielding reconstructions with heterogeneous resolution in part due to the averaging over many conformations. The relative local resolutions are displayed for each target, colored blue to red (Figure 2). The absolute ranges are provided in the distributions (Figure 3A).

### 2.3 RMSF of Predictions Versus Resolution

For each CASP group, the root mean‐square fluctuation (RMSF) for each Cα or C4′ atom was computed from five predictions submitted for a given target. This was performed by first aligning models 2–5 against model 1 using least‐square fit in ChimeraX [16] using the *align* command. The RMSF for each residue was calculated by computing the root mean‐square fluctuation of the Cα atoms from the 5 aligned models.

Only groups where all five predictions had lDDT scores greater than 0.7, and the TM and IPS (in the case of multimers) scores were greater than 0.8, were included in our assessment. We then calculated Pearson's correlation (PCC) between the RMSF and the local resolution of all maps for which the predictions met the accuracy criteria.

### 2.4 Local Accuracy Estimates Versus Resolution

The Local Distance Difference Test (LDDT) [17] is a measure of local agreement between structural models while ignoring the long‐range discrepancies that negatively affect other scores such as RMSD and TM. LDDT scores are typically reported for each residue on a range of 0–1.0 (or as a percentage), with values close to 1.0 indicating a similar local environment. The predicted Local Distance Difference Test (pLDDT), which was first used in AlphaFold2 [11] attempts to reproduce the LDDT scores and thus is an estimate of local model accuracy. Although adopted as standard since CASP15, other estimates of model accuracy also exist, such as the RosettaFold estimate of positional error (the smaller the better) [18]. To deal with the directionality of different reported accuracy estimates, we use the absolute Pearson's Correlation in our analysis.

### 2.5 Local Fit to Density Analysis

In recent CASPs, we have noticed potential modeling errors in experimental models submitted as cryo‐EM targets. Such errors are typically small and unsurprising given the sheer size of many of these targets. Previously, such errors have been apparent during visual assessment of the predictions against the experimental data or when comparing the goodness‐of‐fit of the experimental model against cryo‐EM refined predictions. Although such small errors are not expected to impact the overall ranking of predictions, they do highlight the potential usefulness of structure prediction in the model‐building process.

In previous CASP rounds, the SMOC score [19] was used to assess the goodness‐of‐fit of experimental models, predictions, and refined predictions to the 3D cryoEM map. One of the important features of SMOC and similar local fitness scores, such as Qscore [20] is that they indicate which parts of the model fit the experimental data well. However, this is also a weakness when assessing structure predictions, which may be locally well modeled but, due to some deviations in domain or secondary structure element positioning (e.g., orientation or shifts), only partially fit the experimental data. In contrast, scores such as lDDT are designed to be sensitive to local modeling errors and forgiving to global deviations such as the one described.

To this end, a new localized cryo‐EM fitness score was developed based on aligning fragments to the target, thus avoiding penalties for poor positioning while enabling assessment of local fit to cryo‐EM data. The fragments were computed as sliding windows of 11 residues over each chain of the predictions [19]. SMOC scores of the fragment and the corresponding target residues were then computed. The ΔSMOC score was defined as the difference between the fragment and target SMOC scores. Positive ΔSMOC scores correspond to cases where predicted fragments fit the experimental data better than the target, whereas negative scores indicate the opposite. In the case of high‐resolution targets where sidechain density is well resolved, a more sensitive variant of the above was used with fragments of 5 residues in length. To better disambiguate different modeling errors at high resolutions, SMOC scores were computed on the backbone (backbone SMOC) and sidechain (sidechain SMOC) atoms of the central residue in the fragment.

## 3 Results

### 3.1 RMSF of Predictions Versus Resolution

**FIGURE 4:**
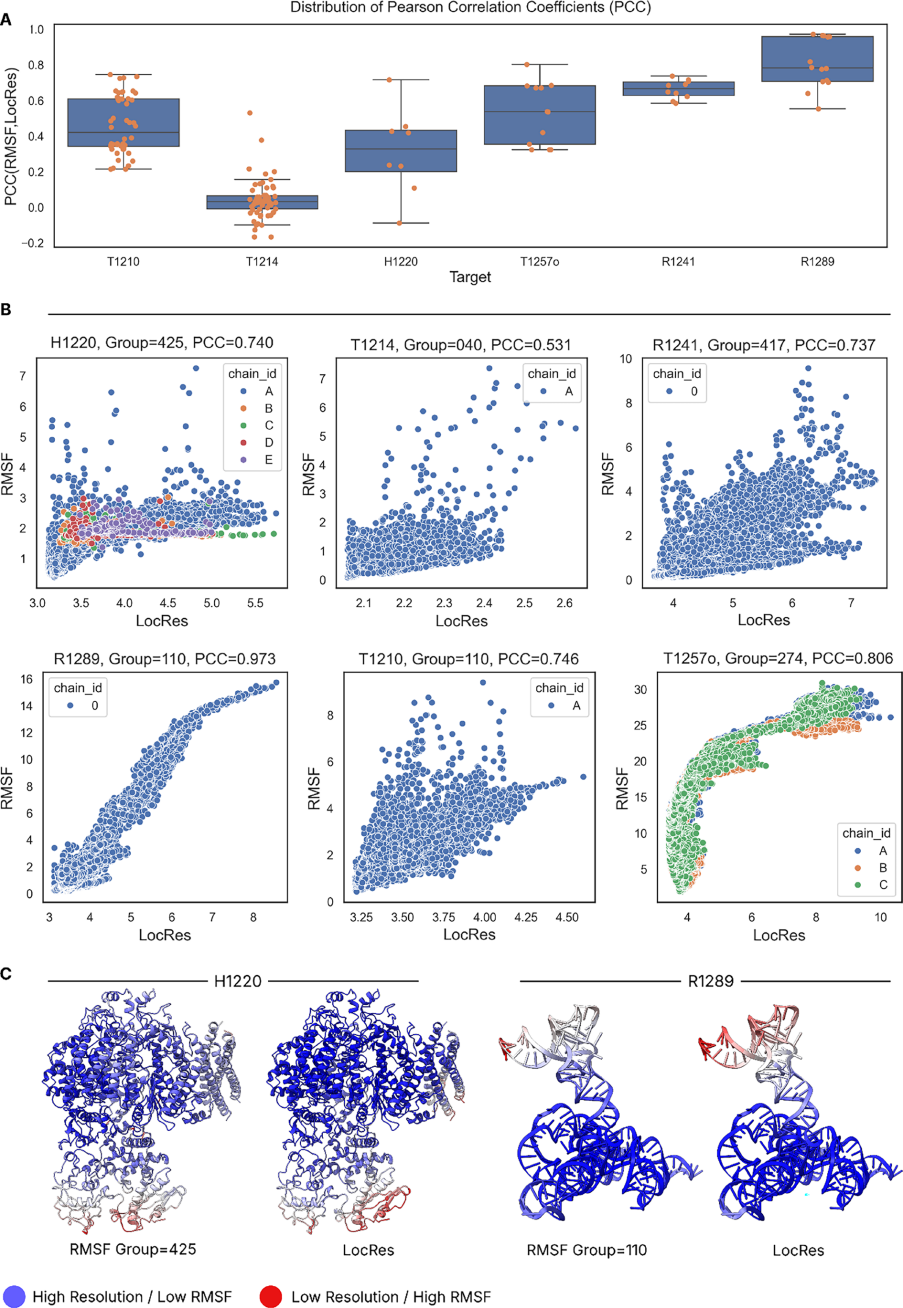
Examples of highly correlated predictions. (A) Distribution of Pearson's correlation coefficients (PCC) of the group RMSF versus LocRes for targets with accurate predictions. (B) RMSF versus LocRes for groups with the highest PCC. Each point is a residue in the model. (C) RMSF and LocRes values for a vitellogenin protein (H1210, PDB: 9ENR) and Group I intron precursor tRNA (R1289).

As stated in methods (Section 3.3), the root mean square fluctuation (RMSF) was computed on well‐predicted targets (LDDT > 0.7 for monomers, > 0.8 and IPS > 0.8 for multimers). Six targets satisfied these accuracy criteria. The Pearson's correlation coefficient (PCC) between the RMSF and local resolution for each of these targets is shown in Figure 4A. Positive correlations were observed, with five targets achieving correlations higher than 0.6 for specific targets (Figure 4B).

The most striking example is of the target R1289, a group I intron precursor RNA, where many groups submitted predictions for which the PCC was close to 1.0, with an average of 0.8. The structure consists of two domains, the intron which makes up the largest mass and is solved at a high resolution, and the tRNA component, which is solved at lower resolution likely due to its moving with respect to the intron (Figure 4C). These resolution differences nicely correlate with the RMSF, reflecting uncertainty in the relative positions of these two regions in predictions. Another impressive example of high correlation between RMSFs and local resolution is the Borna virus replication complex (target H1220) (Figure 3A). This dynamic target is explored in more depth in this issue [21]. The RMSF of predictions for T1214 did not correlate as strongly with local resolution. This may reflect a general lack of flexibility in this target, which had one of the narrowest standard deviations in local resolution (0.08 Å).

### 3.2 Local Accuracy Estimates Versus Local Resolution

**FIGURE 5:**
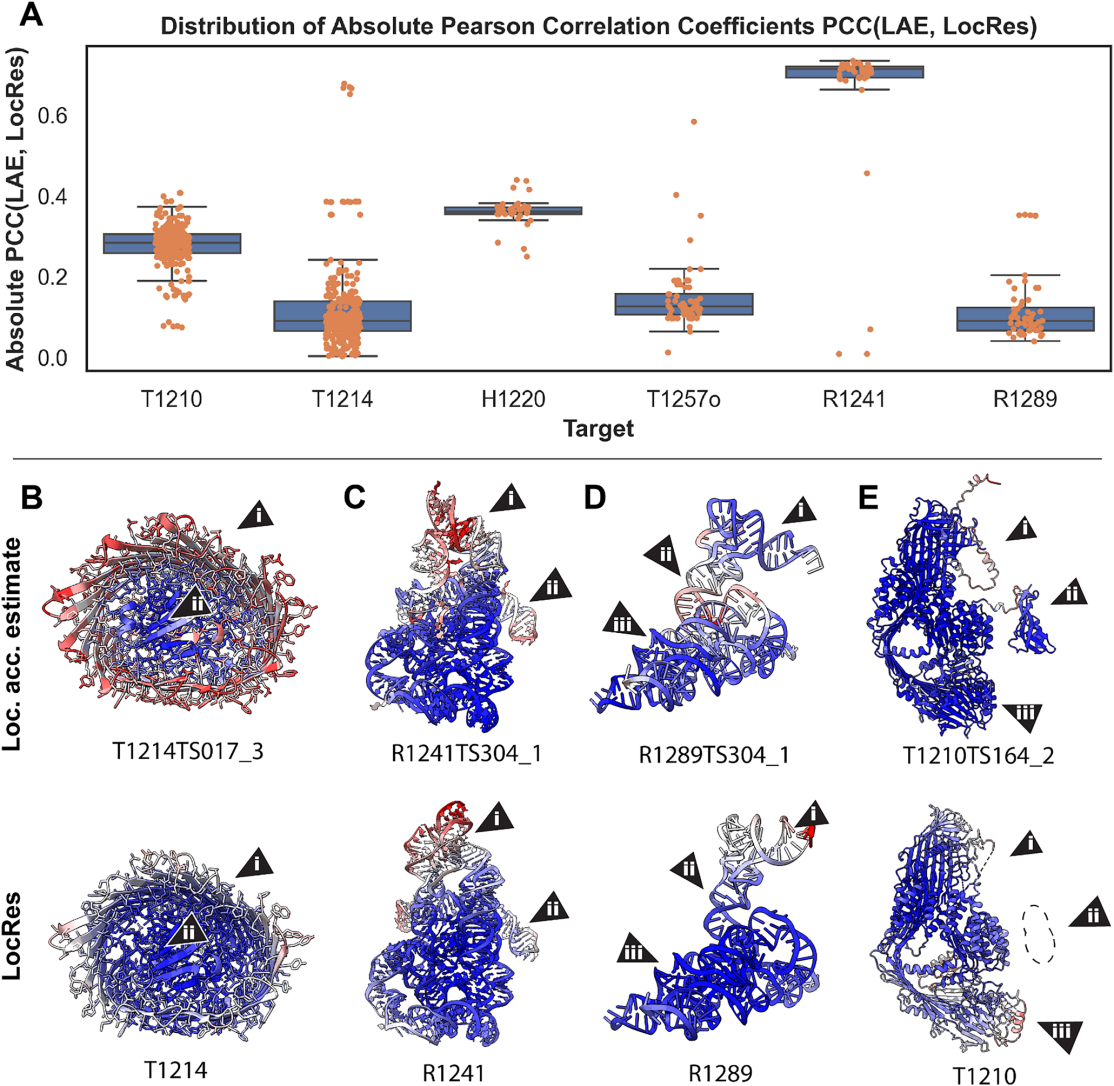
Absolute Pearson's correlation of local accuracy estimates (LAE) versus local resolution. Top panel (A) the distribution of absolute PCC of local accuracy estimates versus local resolution for six targets with accurate predictions. Bottom panel: Examples of different predictions colored by local accuracy estimates (first row) and local resolution mapped on the target (second row). (B) T1214. The model with the highest PCC is shown. The flexible loops, which connect the strands of the beta‐barrel (i), had a lower pLDDT scores than the core (ii), mirroring the local resolution. (C) R1241. The model with the highest absolute PCC missed the interactions between Domains 6 and 2 (i), but accurately recreated Domains 1–5 including flexible regions (ii). (D) R1289. The accuracy‐resolution correlation was poor as most predictors gave the T‐arm and acceptor stem (i) and intron regions (iii) high‐confidence, whereas the D‐arm region (ii) low‐confidence, as exemplified by the AF3 model pictured. (E) T1210. A large unresolved region was given low pLDDT score (i). A region with high estimated accuracy (ii) in the predictions was not visible in experimental data due to being connected to a flexible linker. A large part of the protein had lower resolution likely due to flexibility of the protein but received high pLDDT scores (iii).

Here we study how well local accuracy estimates (LAE) in models correlate with resolution fluctuations in experimental data. For targets with accurate predictions, we observed lower correlation with the experimental data, compared to that of RMSF (see above). Only two out of the six targets had some predictions with LAE‐LocRes PCC values above 0.6 (Figure 5a). T1214 is a TonB‐dependent transporter of Pyrroloquinoline quinone (PQQ) from *E. coli* [22], a large beta‐barrel‐like membrane protein, whereas R1241 is a group‐IIC intron from *O. iheyensis* .

For T1214 (Figure 5B), which only had five high‐correlation predictions, all from group Seder2024hard, the exterior loops that connect the beta‐strands are of low‐resolution (Figure 5Bi), whereas the rest of the structure (Figure 5Bii) has high‐resolution and high accuracy estimates.

In the Group‐IIC intron case, R1241, five out of six domains have been well studied and have structures in the PDB. These five domains had the highest local resolution in the cryo‐EM reconstruction. Domain 6 makes non‐canonical contacts with Domain 2, which were only correctly predicted in 4 Models: 3 from Vfold and 1 from GeneSilico, explored in more detail in the CASP16 RNA assessment paper [23]. Intriguingly, these three accurate Vfold predictions had the lowest correlation with local resolution. Reporting local accuracy estimates for RNA was not mandatory in CASP16, and the GeneSilico group did not provide them. The high correlation between the rest of the predictions and local resolution is thus likely related to the uncertainty in the modeling of Domain 6. An example of a prediction with high correlation but incorrect Domain 6 contacts is shown in Figure 5C.

R1289, the Group I intron precursor tRNA, which had the highest correlation predictions according to RMSF, fared less well against accuracy estimates. Predictors gave high accuracy estimates for the tRNA T‐arm and acceptor stem regions (Figure 5Di), and the group I intron domains (Figure 5Diii), whilst the D‐arm was given lower accuracy estimates (Figure 5Dii). The highest resolution region was the Group I intron domain, which made up the bulk of the structure, whilst the tRNA region, particularly the T‐arm, was solved at a lower‐resolution. Although the pLDDT scores did not correlate well with resolution, the low pLDDT scores of the D‐arm reflect its flexible nature.

Low pLDDT can indicate disordered regions that may not be visible in cryo‐EM reconstructions. Predictions for T1210, a vitellogenin protein from the honey bee, *A. cerana* [24], illustrate two such scenarios. In the first case (Figure 5Ei), a loop with pLDDT scores below 60 was not visible in the experimental data. In the second case (Figure 5Eii), a high‐confidence C‐terminal CTCK domain is predicted but was not visible in the data. This is likely due to its flexible attachment to the bulk of the protein via a disordered region. The information from the pLDDT could explain these discrepancies between prediction and experimental map and model, but did not correlate well with the resolution variation seen at the ends of the model (Figure 5Eiii).

### 3.3 Modeling Errors in Target Structures

**FIGURE 6:**
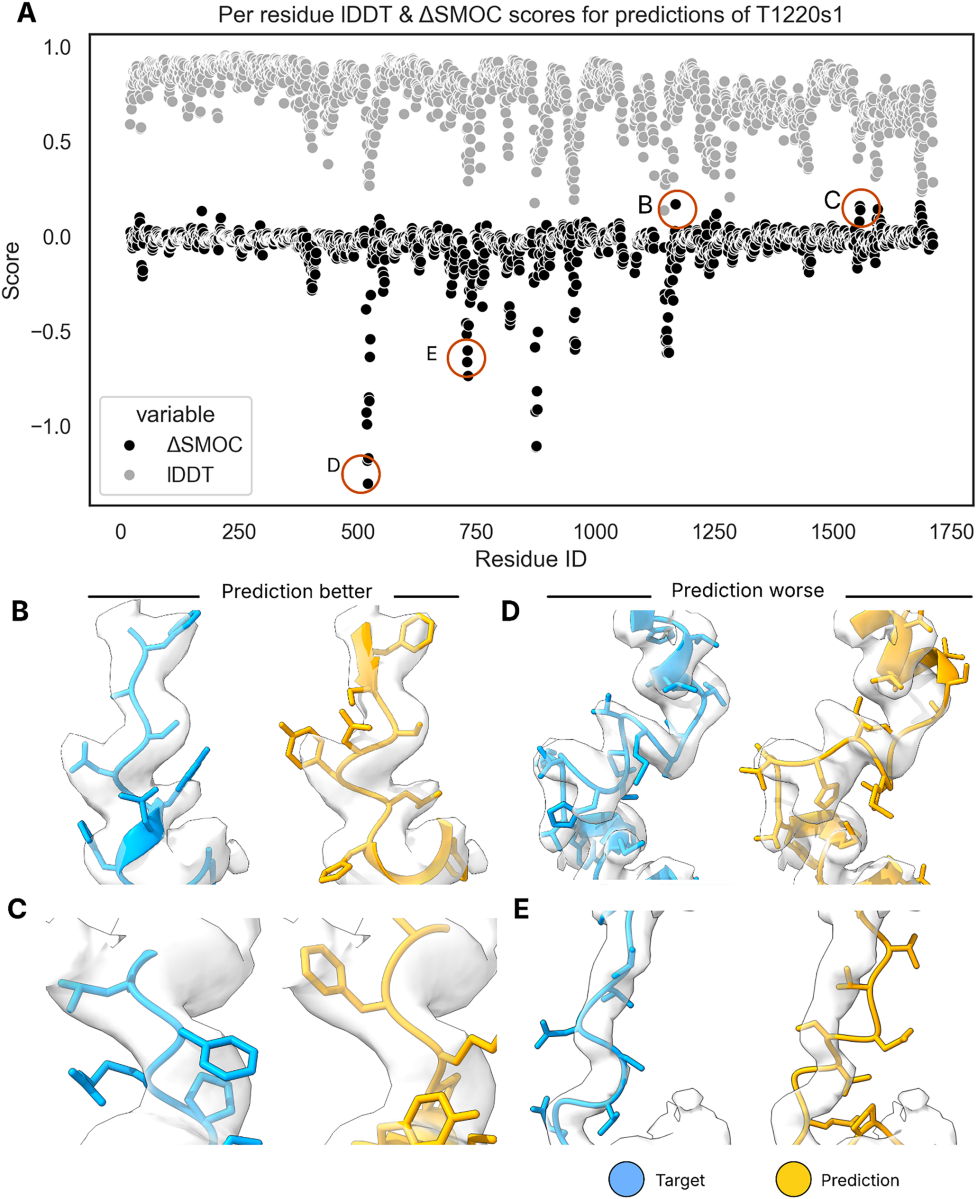
Modeling errors. (A) The local prediction quality, as scored against the reference structure using lDDT (gray) and against the experimental data using ΔSMOC (black), was in good agreement, with low lDDT scores corresponding to low ΔSMOC scores. However, there were some cases where lDDT scores dipped, but ΔSMOC increased. Two such regions are shown in (B) and (C) for the target (blue) and a prediction from group 015 (orange). Despite surpassing the target structure in accuracy in these regions, there were many more counterexamples where predictions struggled to produce locally accurate models, as seen in the many dips in lDDT and ΔSMOC scores. Two such examples are given in panels (D) and (E).

In CASP, the target sequence is sometimes provided before the corresponding structure is finalized. Occasionally, challenging targets are not solved in time, and predictions can expedite the process [12]. In this CASP, a few targets had small errors in the submitted experimental model. These errors were identified by comparing the fit of prediction and experimental model to the experimental data using ΔSMOC scores. Assessment against the experimental data using ΔSMOC produced residue scores which followed the same trends as the lDDT, as exemplified by predictions of the Borna Virus polymerase L‐protein (target T1220s1), which is part of the larger Borna virus replication complex (target H1220) (Figure 6A). However, in some cases where the lDDT score dipped, the ΔSMOC increased, indicating that although the prediction did not agree with the target structure in this region, it better agreed with the experimental data. Examples from the PEZY Folding (group 015) are visualized in Figure 6B,C. We want to critically note here that although many predictors were able to model some loops better than in the experimental model, they could not do this systematically for all loops, as shown in Figure 6D,E.

### 3.4 Side Chain Analysis

**FIGURE 7:**
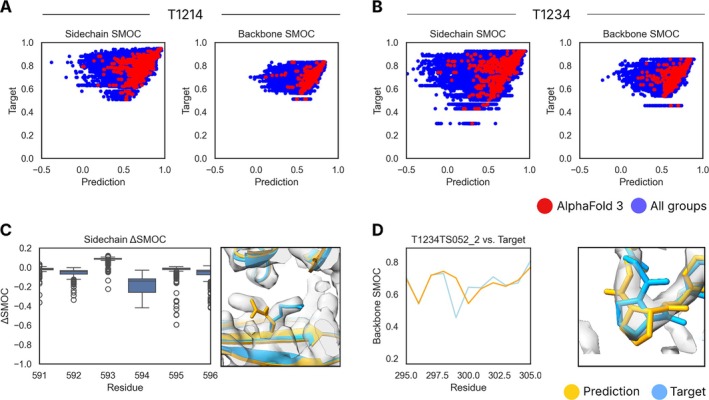
Sidechain and backbone SMOC analysis of high‐resolution protein targets. Sidechain and backbone SMOC scores were plotted for all residues of all predictions versus target for T1214 (A) and T1234‐D1 (B). Despite backbone SMOC scores of predictions generally matching those of the target, the sidechains generally fitted less well. In (C), R594 was an example of a residue from T1214 which had highly variable sidechain SMOC scores. An example of a predicted sidechain is shown. In (D), an example of a backbone region which was better modeled by many predictors. The proline at 299 was *trans* in the target, but predictions such as the one shown were modeled as *cis*.

In CASP15, one target was of sufficiently high resolution that we could directly assess the fit of sidechains against the experimental data. In this CASP, two such targets were present: T1214 and H1236. Given the resolution, we assessed the fit of sidechains and backbone regions of the experimental model and predictions (Figure 7A,B, respectively). Residues with a local resolution worse than 2.5 Å were excluded from these calculations.

Similar to the last CASP, the backbone was generally well predicted, with SMOC scores of predictions approaching those of the experimental model. For sidechains, there was more variability in the SMOC scores, with predictors frequently producing sidechains not fitting the experimental data. The residue R594 was one of many poorly predicted sidechains in T1214. This can be seen in the wide ΔSMOC score distribution for this residue in Figure 7C.

One surprising outlier was a proline residue 299 in target T1234‐D1 (an assessment unit from H1236), which frequently had better fit to the backbone density in predictions than in the provided experimental model (Figure 7D). On closer inspection, the proline in the experimental model was modeled in the more common *trans* conformation, but the predictions, which fitted the density best, were in the *cis* conformation. Such an error is unlikely to affect overall rankings, but highlights how even at relatively high resolutions, modeling errors can be made, which can potentially be avoided with the use of accurate structural predictions. The perhaps surprising ability for AlphaFold and current state‐of‐the‐art methods to predict *cis* prolines has been noted elsewhere [25].

## 4 Discussion and Conclusion

### 4.1 Discussion

Our analysis underscores the increasingly reciprocal relationship between cryo‐EM data and structure prediction in the CASP framework. Although the incorporation of cryo‐EM data into CASP already provides a valuable means of evaluating prediction accuracy, our results highlight how predictions can also illuminate the strengths and limitations of experimental reconstructions. This dual perspective is particularly important given the growing reliance on structure prediction for experimental model building and validation.

This relationship is particularly pertinent today, with high‐confidence models being used to build models into maps by rigid docking. With insufficient structural details from experimental data for further refinement, such models would be problematic for CASP as predictions would be compared against predictions. In this CASP, the target H1272 partly raises this issue. Regions of the reconstruction have sufficient resolution to resolve beta sheets, whilst other regions can only delineate domains. The structure was solved using a combination of model building and rigid docking of AlphaFold2 and AlphaFold3 predictions in low‐resolution regions that are able to provide important biological and molecular insights [26]. But many parts of the experimental model have been solved at an insufficient resolution to be considered a reliable ground truth for assessment of predictions.

A central finding of this study, although based only on 6 targets for which the predictions passed a certain accuracy threshold (lDDT > 0.7 and TM and IPS (for complexes) > 0.8), is that the variability observed across multiple predictions, quantified as RMSF, correlates with cryo‐EM local resolution. This suggests that ensembles of predictions inherently capture conformational heterogeneity, which cryo‐EM maps often reflect in poorly resolved regions. These results align with earlier work showing that molecular dynamics fluctuations correlate with AlphaFold confidence metrics [27]. However, this extends beyond that, indicating that prediction variability is an informative measure of uncertainty that could complement existing model quality metrics in CASP. It is also worth considering that predictors were not asked to submit five models with an RMSF that would correlate with local resolution. Some groups may have favored submitting diverse models or employed other strategies that would have underperformed in this analysis. The degree of diversity among models, and the sampling of diversity, is also an important factor and something that has been a common theme in AlphaFold era CASP experiments including, for example, MassiveFold in this CASP [28].

pLDDT has been shown to correlate only weakly with B‐factor [29] and RMSF from MD simulations [27]. Part of the reason could be that well‐predicted domains are assigned high pLDDT scores even if their orientation with respect to one another is not well defined. At the same time, low pLDDT scores have also been shown to be good indicators of flexible or disordered regions, which may be unresolvable in cryo‐EM experiments. Although less correlated with local resolution variations than RMSF, it remains a valuable metric in its own right for model building and for assessment of predictions including interfaces [30]. Removing low‐confidence regions based on pLDDT values or clustering residues in domains based on PAE matrices is an important step in many downstream docking and refinement procedures [31, 32, 33]. Approaches to combine structure prediction with experimental data such as Phenix PredictAndBuild [32], which iteratively updates input templates for AlphaFold2 by fitting to experimental data, or ROCKET [34] which biases OpenFold's [35] evolutionary space, also exist. Given the importance of local accuracy metrics to these approaches, we hope to see more such methods, particularly in the RNA prediction space, whether in the form of pLDDT or positional error scores.

These results also have implications for assessment methodology. Because predictors often show greater variability in regions of low cryo‐EM resolution, scoring functions that account for resolution‐dependent reliability may provide fairer benchmarks. Similarly, ensemble‐based assessment strategies could help capture conformational dynamics that are otherwise obscured in single static references. Complementary use of prediction‐derived uncertainty measures, such as PAE matrices or distograms [36], may further enrich future evaluations by explicitly linking prediction confidence to underlying conformational flexibility.

Our benchmarking further revealed that high‐quality predictions can help identify errors in experimental reference models. In several cases, predictors consistently disagreed with local regions of deposited structures that were later shown to be problematic. Fragment‐based analysis with ΔSMOC provided a sensitive method for capturing such discrepancies, outperforming manual inspection in both efficiency and precision. This illustrates the utility of prediction‐informed evaluation not only for assessing participants but also for improving experimental models themselves. Incorporating automated ΔSMOC‐style pipelines into CASP could therefore support both communities, ensuring that experimental models for targets reflect the highest possible quality.

One important limitation of the local analyses of predictions against experimental data is that it relies on an experimental model for fitting fragments of predictions to the data. The assumption is that the experimental model is of sufficient quality to make accurate fragment alignment possible. In the cases where there were small modeling errors in the experimental model, this approach was able to identify them by comparing the fit‐to‐density. This may, in part, be due to the relatively wide fragment width of 11 residues. Alternatively, docking using secondary structure or a larger rigid body defined by tools such as Slice'N'Dice [33] or RIBFIND [8] might be an alternative way to find well‐fitting regions of predictions.

Looking ahead, our findings highlight the important interplay between experimental and computational methods. Structure predictions are now indispensable starting points for cryo‐EM, crystallography, and hybrid approaches, accelerating model building while also enabling the detection of errors in deposited structures. At the same time, the availability of high‐quality experimental data remains crucial for assessing the limits of prediction algorithms and for ensuring that benchmark targets are biologically informative. Strengthening this reciprocal exchange through continued provision of experimental data, resolution‐aware evaluation, and systematic pipelines for reporting discrepancies will allow CASP to remain a unique forum for driving both predictive accuracy and experimental rigor.

Uncertainty information in the form of RMSF, local accuracy estimates, or measures such as PAE or distogram information (not explored in this paper) could in principle act as a data‐independent proxy for map interpretation. This may be useful to (i) prioritize regions for focused refinement/model rebuilding, (ii) weight restraints in real‐space refinement, (iii) flag likely alternative conformations and inform reconstruction approaches, and (iv) potentially help in designing cryo‐EM experiments for challenging molecules.

### 4.2 Conclusions

Because structure predictions have become essential starting points for model building in cryo‐EM, crystallography, and lower‐resolution approaches such as cross‐linking mass spectrometry [37], the provision of corresponding experimental data is critical for evaluating and extending their applicability to downstream pipelines. We therefore recommend that structure providers make the experimental data (e.g., half‐maps) available whenever possible. High‐quality predictions can then be used not only for benchmarking but also for detecting and correcting structural errors. In future CASPs, we envision offering automated reporting facilities based on the ΔSMOC method introduced here, to help ensure that reference models are as accurate and informative as possible.

Our analysis further shows that the RMSF across several targets with high‐quality predictions can be an indicator of local resolution variation in the experimental data itself. This indicates that even when accurate models deviate slightly from a single reference, they may still reflect biologically relevant conformational states.

Together, these findings emphasize the reciprocal value of combining experimental data with prediction ensembles, both for assessing predictive accuracy and for improving experimental models.

## Author Contributions

**Thomas Mulvaney:** conceptualization, methodology, software, data curation and analysis, investigation, validation, visualization, resources, manuscript writing, reviewing, and editing. **Andriy Kryshtafovych:** data curation, resources, writing – review and editing, funding acquisition. **Maya Topf:** conceptualization, methodology, analysis, investigation, writing, reviewing, editing, and funding acquisition.

## Funding

This work was supported by Deutsche Forschungsgemeinschaft SFB (1648/1 2024 — 512741711) and Wellcome Trust Collaborative Award in Science (209250/Z/17/Z), (MT and TM) and by the NIH/NIGMS grant R01GM100482 (AK).

## Conflicts of Interest

The authors declare no conflicts of interest.

## Supporting information


**Table S1:** Summary of structures and experimental data.

## Data Availability

Predictions are available at CASP16's official website: https://www.predictioncenter.org/casp16/index.cgi. Processed data, materials, and code are available upon request.
