# Supplementary material for: Cryo‐EM Analysis in CASP16
Source: Proteins. 2025 Dec 11;94(1):447–59. doi: 10.1002/prot.70099 (PMC12750033; doi:10.1002/prot.70099)
Supplement: Supplementary file 1 — Table S1: Summary of structures and experimental data. [file PROT-94-447-s001.pdf]

Supplemental material for Cryo-EM analysis in CASP16

Thomas Mulvaney[1,2], Andriy Kryshtafovych[3], Maya Topf[1,2,4]

Table S1: Summary of structures and experimental data

**Type**	**Target**	**Resolution**	**Resolution StdDev**
Protein	T1210	3.52	0.21
	T1214	2.17	0.08
	H1220	3.54	0.45
	H1229	3.48	0.13
	H1230	3.08	0.09
	H1236	2.39	0.05
	T1257o	4.59	1.59
	H1258	4.92	0.98
	T1270o	3.86	0.36
	H1272	6.47	3.13
	Protein Avg.	3.80	0.71
RNA	R1241	4.30	0.68
	R1242	3.06	0.23
	R1248	3.22	0.32
	R1251o	4.30	0.40
	R1252o	4.96	0.56
	R1253v1	6.59	1.07
	R1253v2	7.02	1.35
	R1254	6.57	0.56
	R1255	9.9	1.56

	R1256	18.51	0.66
	R1281o	9.82	0.71
	R1283v1	6.11	1.36
	R1283v2	6.94	1.44
	R1283v3	10.17	1.38
	R1285o	3.05	0.15
	R1286	7.08	0.95
	R1289	3.91	0.98
	RNA Avg.	6.80	0.85
Hybrid	M1212	3.24	0.36
	M1221	5.00	2.98
	M1224	2.69	0.55
	M1228v1	6.85	1.07
	M1228v2	7.32	0.90
	M1239v1	5.97	0.70
	M1239v2	6.72	0.94
	M1268	3.32	0.43
	M1271	3.98	1.04
	M1282	3.48	0.21
	M1297	4.53	0.88
	Hybrid Avg.	4.83	0.92
